# The association of multiple metrics for evaluating antimicrobial use in U.S. beef feedyards

**DOI:** 10.3389/fvets.2022.1056476

**Published:** 2023-01-04

**Authors:** Michael D. Apley, Nora F. D. Schrag, David E. Amrine, Brian V. Lubbers, Randall S. Singer

**Affiliations:** ^1^Department of Clinical Sciences, College of Veterinary Medicine, Kansas State University, Manhattan, KS, United States; ^2^Livestock Veterinary Resources, LLC, Olsburg, KS, United States; ^3^Department of Veterinary and Biomedical Sciences, College of Veterinary Medicine, University of Minnesota, St. Paul, MN, United States; ^4^Mindwalk Consulting Group, LLC, Falcon Heights, MN, United States

**Keywords:** antimicrobial use, feedyards, feedlots, antimicrobial use monitoring, antimicrobial use reporting, metrics, antimicrobial metric comparison

## Abstract

In order to accurately portray antimicrobial use in food animals, the need for standardized metrics, and an understanding of the characteristics of different metrics, has long been recognized. Fourteen U.S. feedyards were used to evaluate the effects of using centralized constants such as defined daily dose (DDD) and defined course dose (DCD) applied to the weight of medically important antimicrobials by class (mg) as opposed to using electronic individual animal treatment records and lot level in-feed antimicrobial records obtained from the same population. Three numerators were calculated directly from recorded data for each drug product: the number of antimicrobial regimens associated with indication (Reg), milligrams of drug administered per regimen (mg), and calendar days of administration for each regimen (CDoA). There were four use indications to which numerators were assigned: liver abscess control (LAC), bovine respiratory disease (BRD), lameness (lame), or all other indications combined (other). Three denominators were also calculated directly from the data, these being the number of days animals were present (head days), number of cattle received (head in), and kilograms of live weight sold (kg-LW). Numerators and denominators were calculated at the lot level. The use of DDD or DCD was explored to determine how their use would affect interpretation of comparisons between lots or feedyards. At the lot level across both study years, the lot estimate of nDDD differed from the CDoA value by >25% in 49.2% of the lots. The number of Defined Course Doses (nDCD) was then compared to the number of Regimens (Reg). Comparing nDCD to Reg at the lot level across both study years, the lot estimate of nDCD differed from the Reg value by >25% in 46.4% of lots. Both year and metric were also shown to affect numerical feedyard ranking by antimicrobial use according to seven different metrics. The analysis reported here adds to the body of literature reporting substantial effects of metric choice on the conclusions drawn from comparing antimicrobial use across multiple production sites.

## Introduction

In order to accurately portray antimicrobial use in food animals, the needs for standardized metrics and an understanding of the characteristics of different metrics have long been recognized (1). The current “clear lack of standardization, resulting in poor transparency, and comparability” has been recognized by leaders in the field of food animal antimicrobial use metrics (2).

Antimicrobial use data are not interpreted in a vacuum. Individual food animal production sites are evaluated in comparison to other sites. Countries are also evaluated in comparison to other countries. These interpretations have consequences in relation to the viability of individual food animal producers and also for access to markets and international trade. The OIE Annual Report on Antimicrobial Agents Intended for Use in Animals reports antimicrobial use as a numerator of kg of active compound, reported by one of three methods consisting of varying granularity, expressed over a denominator of animal biomass (3). The OIE “continues to advise caution in the interpretation and use of the quantitative data presented in their reports” (4). Nuances of antimicrobial use data must be considered when attempting to make comparisons between countries or regions, since there may be large variations in animal production, disease incidence, and other factors related to antimicrobial use. Presentation of charts and figures without regard to the underlying data can lead to gross misinterpretation of any country′s situation (5, 6). While one application of antimicrobial use data is public presentation for policy purposes, another is stewardship programs where veterinarians and producers evaluate their use in the context of others. Understanding the effects of using different metrics in each of these cases is of great importance.

In this paper, 14 U.S. feedyards are used to evaluate the effects of metric selection on assessing the use of medically important antimicrobials. Relationships between different calculation methods are presented as correlations between numerators, denominators, and seven metrics consisting of combinations of these numerators and denominators. In addition, the effect of metric selection on feedyard ranking is evaluated, both as simple numerical ranks and as proportional ranks. Included in the metrics evaluated are centralized constants (i.e., the dose definitions DDD, DCD) applied to weight (mg) of medically important antimicrobials used by class in comparison to electronic individual animal treatment records and lot level in-feed records obtained from the same population. The data and analysis reported here are related to a subset of feedyards from a larger set of 20 feedyards for which antimicrobial use metrics are reported in a companion paper (7). This subset was selected for the availability of sufficiently granular data to support the analysis.

## Materials and methods

### Feedyard recruitment

Participating feedyards were recruited through investigator relationships, beef producer organizations and feedyard consulting veterinarians. The data reported here represent a sample of convenience based on willingness to participate and the ability to access antimicrobial use records, and do not represent a random sample of the industry.

### Data collection and management

Methods for collecting and managing the data used in this study have been previously reported (7, 8). Briefly, data were supplied as CSV files provided either directly to the investigators or through intermediaries who routinely collected antimicrobial use data from the feedyards. Data were subjected to a series of quality assurance steps which eliminated some lots of cattle within each feedyard. To be included in the final data set, each lot had to fall within inclusion bounds for multiple parameters designed to exclude inaccurate or non-sensical entries, which may be due to errors in recording. These bounds included an average in-weight of < 682 kg (1,500 lbs), an average live weight at the time of shipment of < 818 kg (1,800 lbs), at least 9 animals per lot, at least 7 days and no more than 365 days on feed, and not missing necessary information for calculation of numerators or denominators such as lot number, number of animals, days on feed or weight at time of shipment. Further quality control practices included checking that the lot number and lot data matched for all data, that there were no duplicate entries for the same lot, that the recorded drug was an antimicrobial, and that the amount administered was plausible.

### Calculation of metrics

A table of abbreviations and calculations is available in the Supplemental material.

The analysis of the relationship between different metrics in this paper is based on feedyard and lot units. Within a feedyard, a lot is a management entity representing a group of cattle, often but not always housed together in the same pen, which remains as an economic and management unit from the time of arrival in the feedyard until the lot is sold (closed out). Each of the 14 feedyards had multiple lots which were sold during 2018 and 2019, with an overall total of 6,460 lots containing 936,660 head of cattle. The number of lots sold by a feedyard in the study period was dependent on their feeding capacity, duration of feeding each lot (dependent on the purchase weight, weight at which the cattle were sold, and the rate of gain achieved in the interim), and market influences.

Three numerators were directly calculated from the data for each drug product: the number of antimicrobial regimens associated with indication (Reg), milligrams of drug administered per regimen (mg), and calendar days of administration for each regimen (CDoA).

There were four use indications to which numerators were assigned, consisting of liver abscess control (LAC), bovine respiratory disease (BRD), lameness (lame), or all other indications combined (other). Route of administration was also recorded. In-feed was the only route for LAC. Use for the indication of BRD consisted of in-feed for both treatment and control, injectable for control, and injectable for individual animal therapy. Uses for the lame and other categories consisted of only individual animal injectable therapy. Numerators were calculated at the lot level individually for each drug product. A total numerator value for a lot or feedyard was calculated from the sum of values for all drug products recorded for that lot or feedyard.

Three denominators were also calculated directly from the data, these being the number of days animals were present (head days), number of cattle received (head in), and kilograms of live weight sold (kg-LW). The summed head days were divided by 365 and expressed as animal years (AY) or expressed as 100 head days in the metrics evaluated here. Denominators were calculated individually for each lot.

The number of defined daily doses (nDDD) and number of defined course doses (nDCD) were calculated for each drug product. For example, a value was calculated for both 200 mg/ml and 300 mg/ml oxytetracycline, and for all 3 formulations of ceftiofur. The defined daily dose (DDD) value was calculated for each drug product by summing all milligrams administered for each drug product across all lots in all feedyards and dividing by CDoA for each drug product summed across all lots in all feedyards, yielding a single value for mg/CDoA. This value is equivalent to the mean dose administered per calendar day of administration across all feedyards and is therefore used as the defined daily dose (DDD) for this study. To calculate the defined course dose value (DCD), the same mg value as for DDD was divided by the sum of all regimens for that drug product across all lots in all feedyards, yielding a single mean value for mg/Reg, which is equivalent to the DCD. These DDD and DCD values were then divided into the sum of the product specific milligrams for a lot or feedyard to determine the nDDD and nDCD, respectively, to be assigned to that lot or feedyard. These same calculations were conducted for each use indication within a feedyard. Note that an estimation of days of exposure for single injection antimicrobials was not used in this study.

The relationship between nDDD and CDoA was evaluated at both the feedyard and lot level by subtracting the nDDD value from the CDoA value and then dividing this difference by the CDoA value to express the difference as a percentage of the CDoA. The differences are presented graphically and categorized by the difference being within 10%, between 10% and 25%, and > 25%. The relationship between nDCD and Reg was evaluated in the same manner; the value for nDCD for a lot or feedyard was subtracted from the Reg value and this difference was divided by the Reg value.

### Statistical analysis

#### Correlations of numerators, denominators, and metrics

Correlations of numerator, denominator, and metric values were analyzed by using the “cor” function in base R (9), and setting the method to “spearman”. The Spearman method was chosen due to concerns that the distributions were not sufficiently normal to utilize Pearson correlations.

#### Evaluation of feedyard rankings by different metrics

The first step in comparing feedyard rankings according to different metrics was to sum, at the feedyard level, the numerator values for all drug products as well as summing all values for each denominator. Each of the metrics were then calculated from combinations of these numerators and denominators for each feedyard. Ranking was performed as both simple ranks based on values using the “minrank” function in R, and by using the “rescale” function in R to rescale each metric to values ranging from 0 to 1. The rescale method preserved the magnitude of differences in numeric values between feedyards, as well as preserved the distribution within metric.

In addition to these rankings, a Wilcoxon rank test was also performed for each possible pair of metrics reported at the feedyard level. The base R wilcox.test function for paired samples was used to calculate a test statistic and *p*-value at a 95% confidence level. This analysis was conducted for all use indication categories combined.

## Results

### Use of nDDD to estimate CDoA, and nDCD to estimate Reg, for individual feedyards and lots

#### Calculated DDD and DCD values

The DCD values used in the analysis are reported in Table 1. Injectable products were all single injection regimens in this dataset and estimated duration of therapy calculations were not utilized to estimate DDD values; therefore, the DDD value is equivalent to the DCD value for these products since the entire dose was administered on 1 day. In contrast, the in-feed antimicrobials tylosin and chlortetracycline were administered each day for multiple days. The CDoA per regimen (mean ± SD) values were 154 ± 58 days for tylosin and 10 ± 10 days for chlortetracycline, yielding a mg/CDoA (DDD) value of 82 and 3,932 mg, respectively. The 350 mg/head per day chlortetracycline regimen for control of BRD, with no limit on duration of administration, accounted for 9.8% of the chlortetracycline regimens reported here. This longer regimen duration, as compared to a maximum of 5 days for the 10 mg/lb per day regimen indicated for treatment of BRD (90.2% of chlortetracycline regimens), had the effect of increasing the mean CDoA value of chlortetracycline to 10 days as compared to the median value of 6 days.

**Table 1:** Calculated DCD values used in the analysis.

**Antimicrobial class**	Antimicrobial product^*^	**Total regimens by product**	**Percent of all regimens**	**Mean calendar days of administration (CDoA)**	**Mean mg per regimen** **(DCD)^†^**
Aminoglycoside	Neomycin (oral individually)	1,714	0.1%	1	7,665
Cephalosporin	Ceftiofur crystalline free acid	60,237	4.8%	1	1,957
	Ceftiofur hydrochloride	2,319	0.2%	1	785
	Ceftiofur sodium	793	0.1%	1	538
Fluoroquinolone	Danofloxacin	7,868	0.6%	1	3,709
	Enrofloxacin	16,632	1.3%	1	2,443
Macrolide	Gamithromycin	7,521	0.6%	1	1,818
	Tildipirosin	6,178	0.5%	1	1,048
	Tilmicosin	14,833	1.2%	1	2,282
	Tulathromycin	99,200	7.9%	1	705
	Tylosin (in feed)	898,265	71.4%	154 ± 58	12,543
	Tylosin	2	< 0.01%	1	8,000
Penicillin	Ampicillin	8,883	0.7%	1	6,717
	Penicillin G procaine	902	0.1%	1	9,705
Phenicol	Florfenicol	18,804	1.5%	1	11,709
	Florfenicol / flunixin meglumine	6,585	0.5%	1	12,638
Sulfonamide	Sulfadimethoxine	381	0.03%	1	736,732
	Sulfamethazine bolus 1 (oral)	30	0.002%	1	115,000
	Sulfamethazine bolus 2 (oral)	1,958	0.2%	1	120,941
Tetracycline	Chlortetracycline (in feed)	45,267	3.6%	10 ± 10	36,374
	Oxytetracycline 200 mg/ml	37,200	3.0%	1	6,352
	Oxytetracycline 300 mg/ml	22,166	1.8%	1	8,853
Total regimens		1,257,738			

The calculated DCD and DDD values differ only for the in-feed drugs (see footnote and text).

^*^Products are injectable except where noted otherwise.

^†^For injectable antimicrobials and those administered orally to individual animals, the mean dose per regimen (mg/Reg) is the same as the mean dose per Calendar Day of Administration (mg/CDoA) because in all cases these antimicrobials were only administered once and only the day of administration is accounted for in Calendar Days of Administration (CDoA); days of therapy are not estimated. For the in-feed antimicrobials, mean mg/CDoA values of 82 for tylosin and 3,932 for chlortetracycline were observed, and were used as DDD values for calculations.

#### Use of nDDD to estimate CDoA

The percent difference between the nDDD and the CDoA, expressed as a percent of the CDoA value, for all indications combined are presented in Table 2. The lot level comparisons are across all lots in both years of the study, while feedyard level data are presented by year.

**Table 2:** The percent difference between the nDDD and the CDoA, expressed as a percent of the CDoA value, for all indications combined.

	**Feedyard level** **(*****N*** = **14)**			**Lot level** **(*****N*** = **6,460)**	
	**Year**	**Feedyard count**	**Percent**	**Lot count**	**Percent**
>25% difference	2018	7	50.0%	3,176	49.2%
	2019	3	21.4%		
>10% to 25%	2018	3	21.4%	1,756	27.2%
	2019	6	42.9%		
Within 10%	2018	4	28.6%	1,528	23.7%
	2019	5	35.7%		

**Figure 1:**
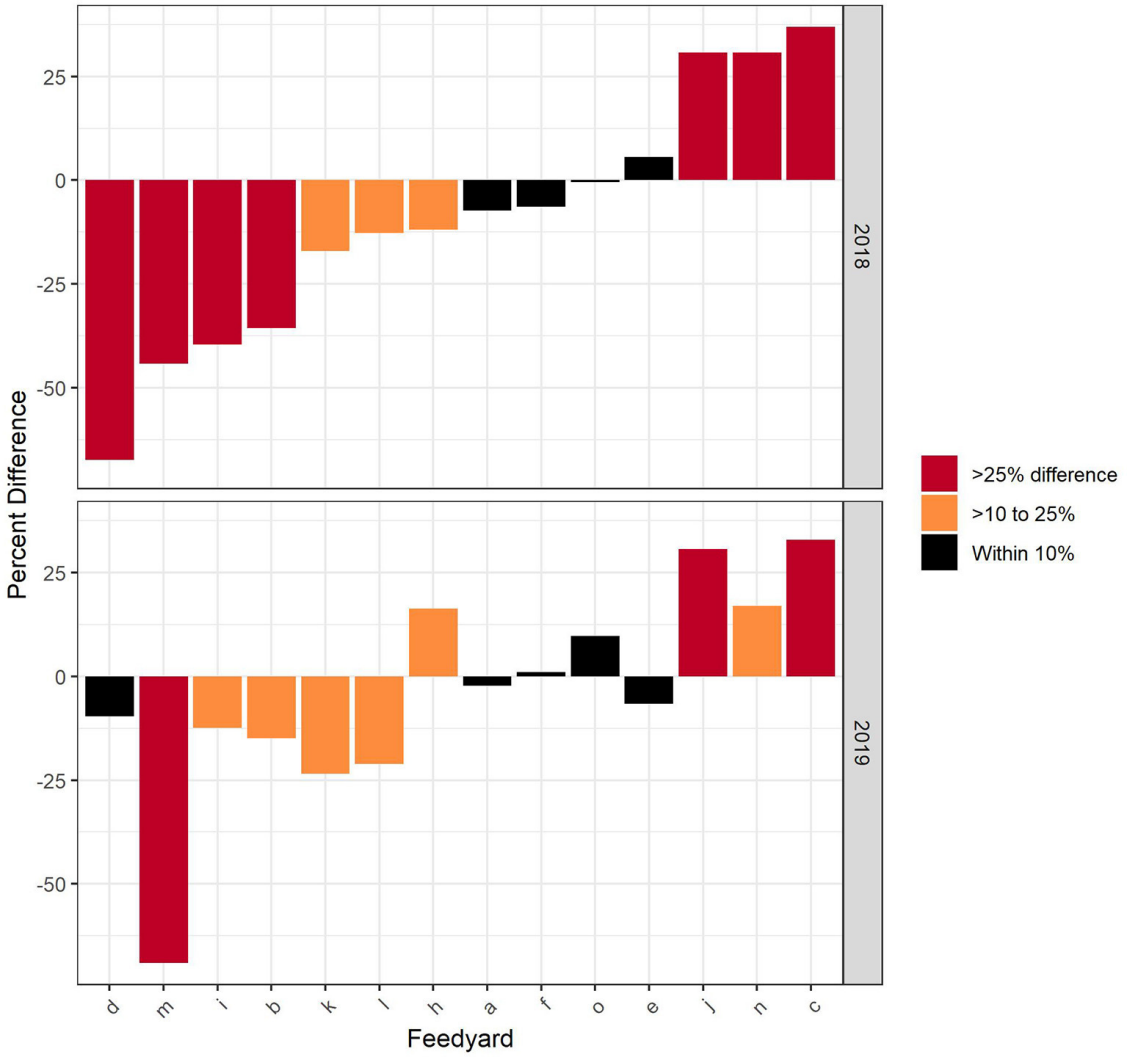
Comparison of CDoA and nDDD at the feedyard level with the difference expressed as the percentage of CDoA for each feedyard.

Comparisons of nDDD and CDoA for each feedyard by year are illustrated in Figure 1. A positive value indicates the CDoA value was larger than the nDDD. Table 2 reports that, considering cattle closed out in 2018, the feedyard estimate of nDDD was within 10% of the CDoA value in 4 (28.6%) of the feedyards, between 10% and 25% in 3 (21.4%) feedyards, and the difference was >25% in 7 (50.0%) feedyards. In 2019, the values for within 10%, > 10% to 25%, and > 25% were 5 (35.7%), 6 (42.9%), and 3 (21.4%), respectively. These comparisons only show the relative difference between nDDD and CDoA. The actual difference may be negative or positive, therefore nDDD does, in fact, both overestimate and underestimate antimicrobial use when compared to actual use data.

**Figure 2:**
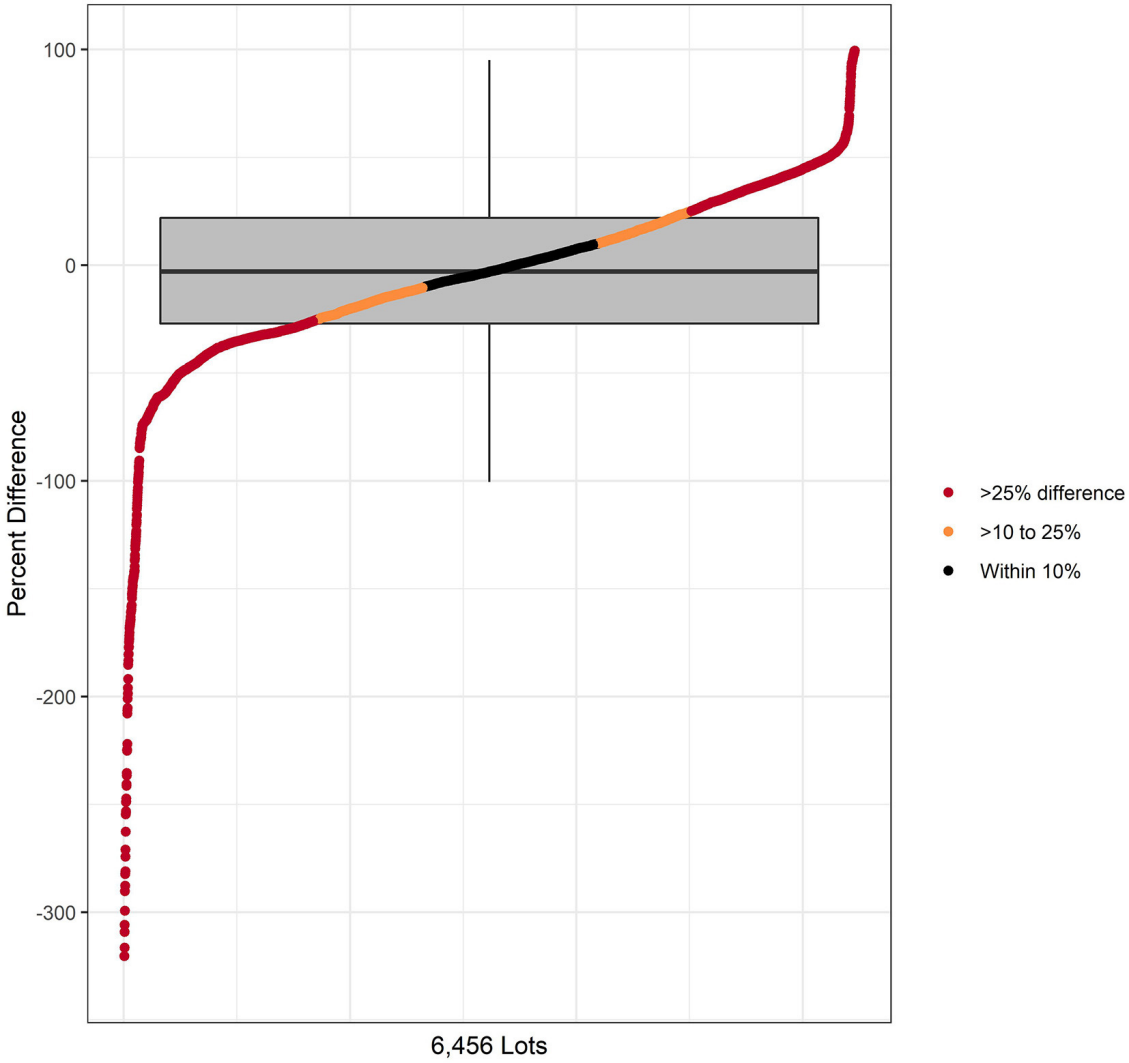
Comparison of CDoA and nDDD at the lot level with the difference expressed as the percentage of CDoA for each lot. The distribution on the X axis represents the values for 6,456 lots after 4 lots were omitted for truncation of the Y axis. Data are reported as both a distribution and as a box and whiskers plot The box and whisker plot corresponds only to Y axis values for percent difference.

When considering cattle closed out across all 6,460 lots for both 2018 and 2019 in Table 2, the lot estimate of nDDD was within 10% of the CDoA value in 23.7% of the lots, > 10% to 25% in 27.2%, and the difference was >25% in 49.2%. In Figure 2, these results are presented for the relationship of nDDD to CDoA as calculated for each individual lot across both study years with the omission of 4 extreme outlier lots to allow truncation of the negative percentage value y-axis at−350%. The distribution of the remaining 6,456 lots is arranged from the most negative percent difference value on the left to the most positive value on the right. The distribution is superimposed on a box and whisker plot where the box contains the middle 50% of values and a line indicating the median value. The whiskers extend beyond the box by 1.5 times the interquartile range (25^th^ to 75^th^ percentile). The median value is−2.9% with the negative whisker extending to a value of−99.3%. The positive whisker extends to a value of 95.1%. Within this range, all but 145 (2.2%, the outliers) of the 6,460 total lots are represented.

#### Use of nDCD to estimate regimens

The percent difference between the Reg and nDCD, expressed as a percent of the Reg value, for all indications combined are presented in Table 3. The lot level comparisons are across all lots in both years of the study, while feedyard level data are presented by year.

**Table 3:** The percent difference between nDCD and Reg, expressed as a percent of the Reg value, for all indications combined.

	**Feedyard level** **(*****N*** = **14)**			**Lot level** **(*****N*** = **6,460)**	
	**Year**	**Feedyard count**	**Percent**	**Lot** **count**	**Percent**
>25% difference	2018	3	21.4%	2,998	46.4%
	2019	3	21.4%		
>10% to 25%	2018	5	35.7%	1,977	30.6%
	2019	5	35.7%		
Within 10%	2018	6	42.9%	1,485	23.0%
	2019	6	42.9%		

**Figure 3:**
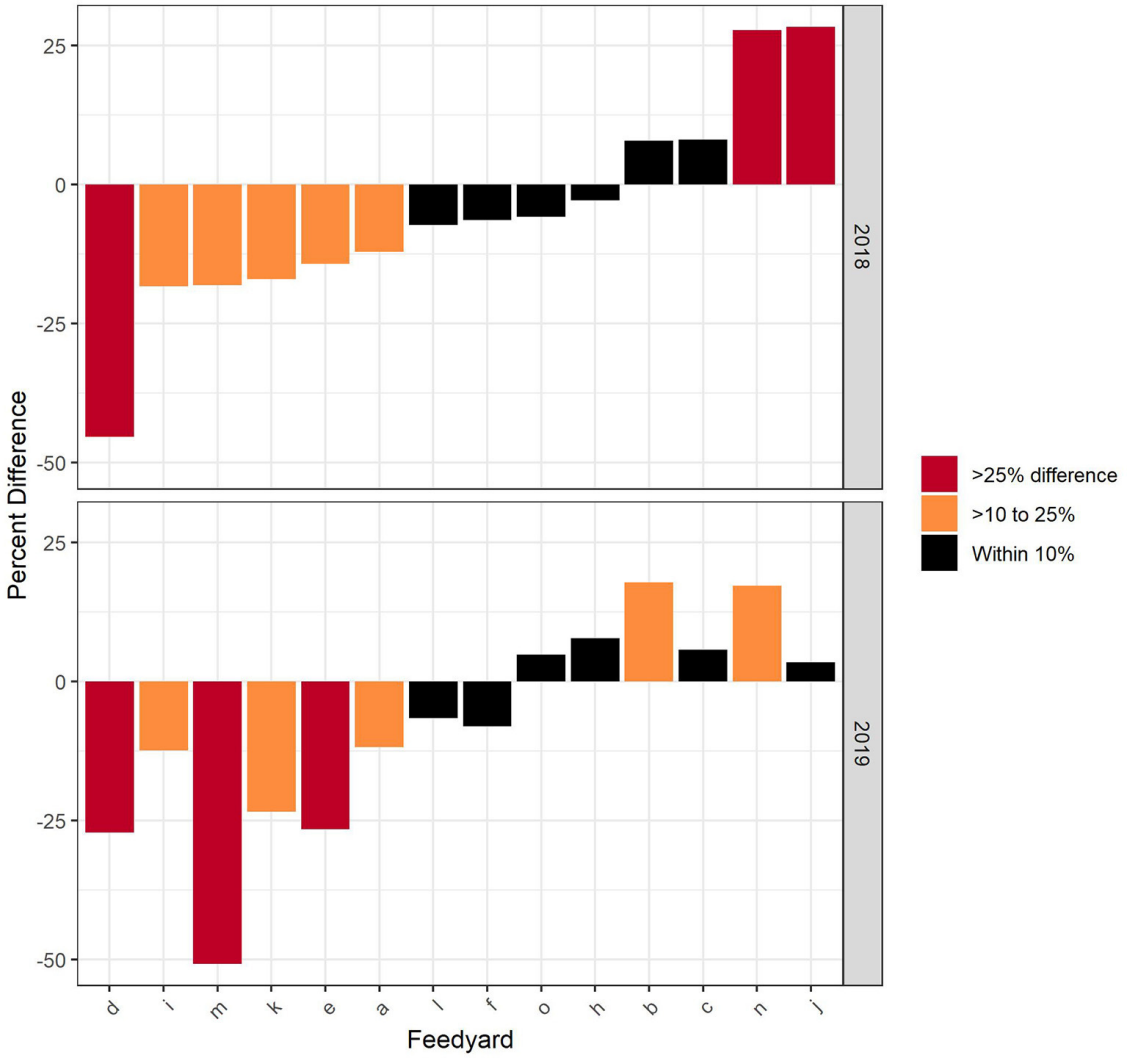
Comparison of Reg and nDCD at the feedyard level with the difference expressed as the percentage of Reg for each feedyard.

The percent difference between the Reg and nDCD values for all indications in each feedyard is presented in Figure 3. A positive value indicates the Reg value was larger than the nDCD. When considering cattle closed out in either 2018 or 2019, the feedyard estimate of nDCD was within 10% of the Reg value in 6 (42.9%) of the feedyards, > 10% to 25% in 5 (35.7%) and displayed >25% difference in 3 (21.4%) of the feedyards.

**Figure 4:**
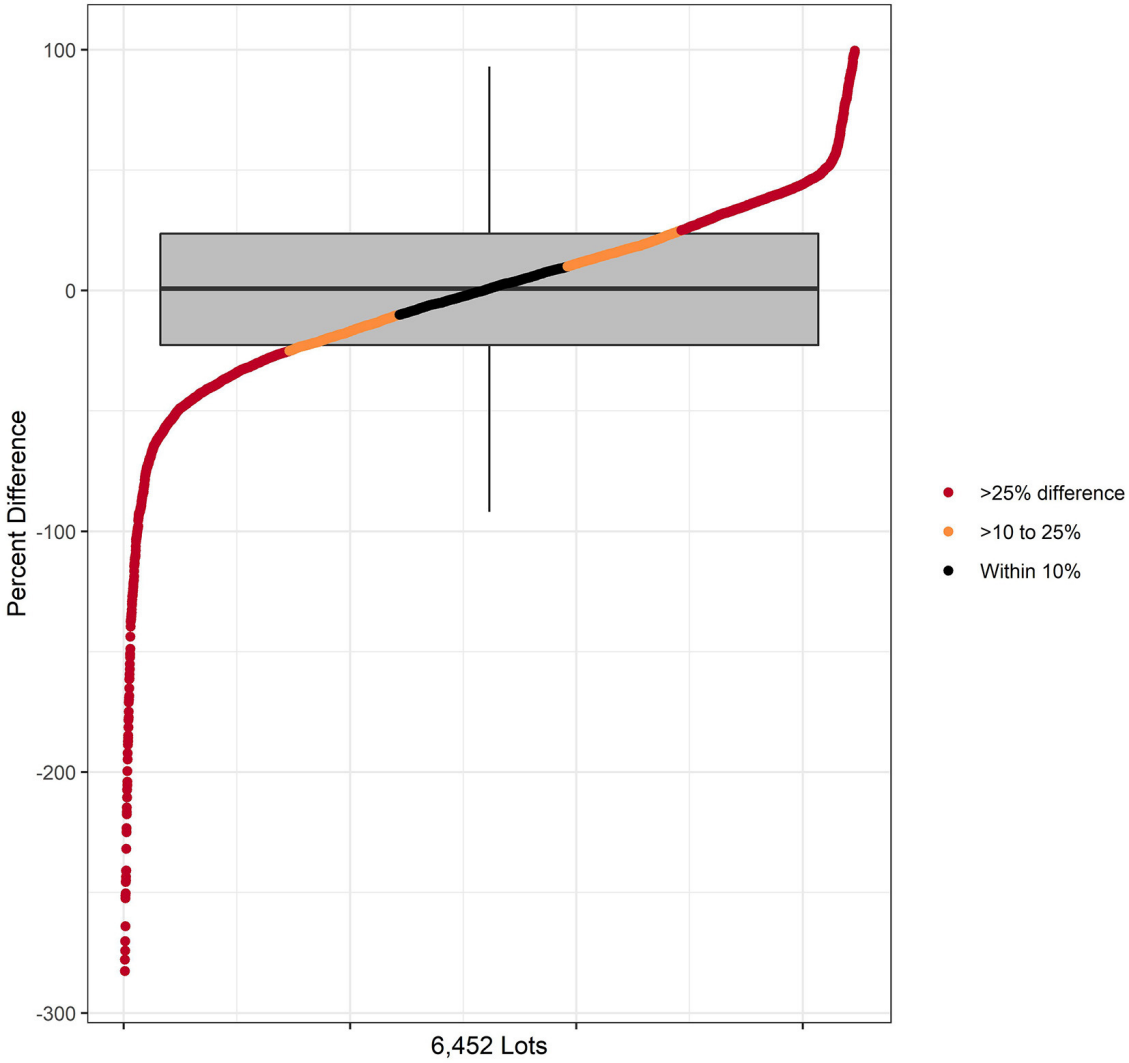
Comparison of Reg and nDCD at the lot level with the difference expressed as the percentage of Reg for each lot. The distribution on the X axis represents the values for 6,452 lots after 8 lots were omitted for truncation of the Y axis. Data are reported as both a distribution and as a box and whiskers plot. The box and whisker plot corresponds only to Y axis values for percent difference.

When considering cattle closed out in all 6,460 lots in both 2018 and 2019, as reported in Table 3 the lot estimate of nDCD was within 10% of the Reg value in 23.0% of the lots, >10% to 25% in 30.6% of the lots, and displayed >25% difference in 46.4% of lots. In Figure 4, these results are presented for the relationship of nDDD to CDoA as calculated for each individual lot across both study years with the omission of 8 extreme outlier lots to allow truncation of the negative percentage y-axis value at−300%. The distribution of the remaining 6,452 lots is arranged from the most negative percent difference value on the left to the most positive value on the right. The median value represented in the box and whisker plot is 0.9% with the negative whisker extending to a value of−91.9%. The positive whisker extends to a value of 93.0%. Within this range, all but 162 (2.5%, the outliers) of the 6,460 total lots are represented.

### Correlation of numerators and denominators

#### Numerators

**Figure 5:**
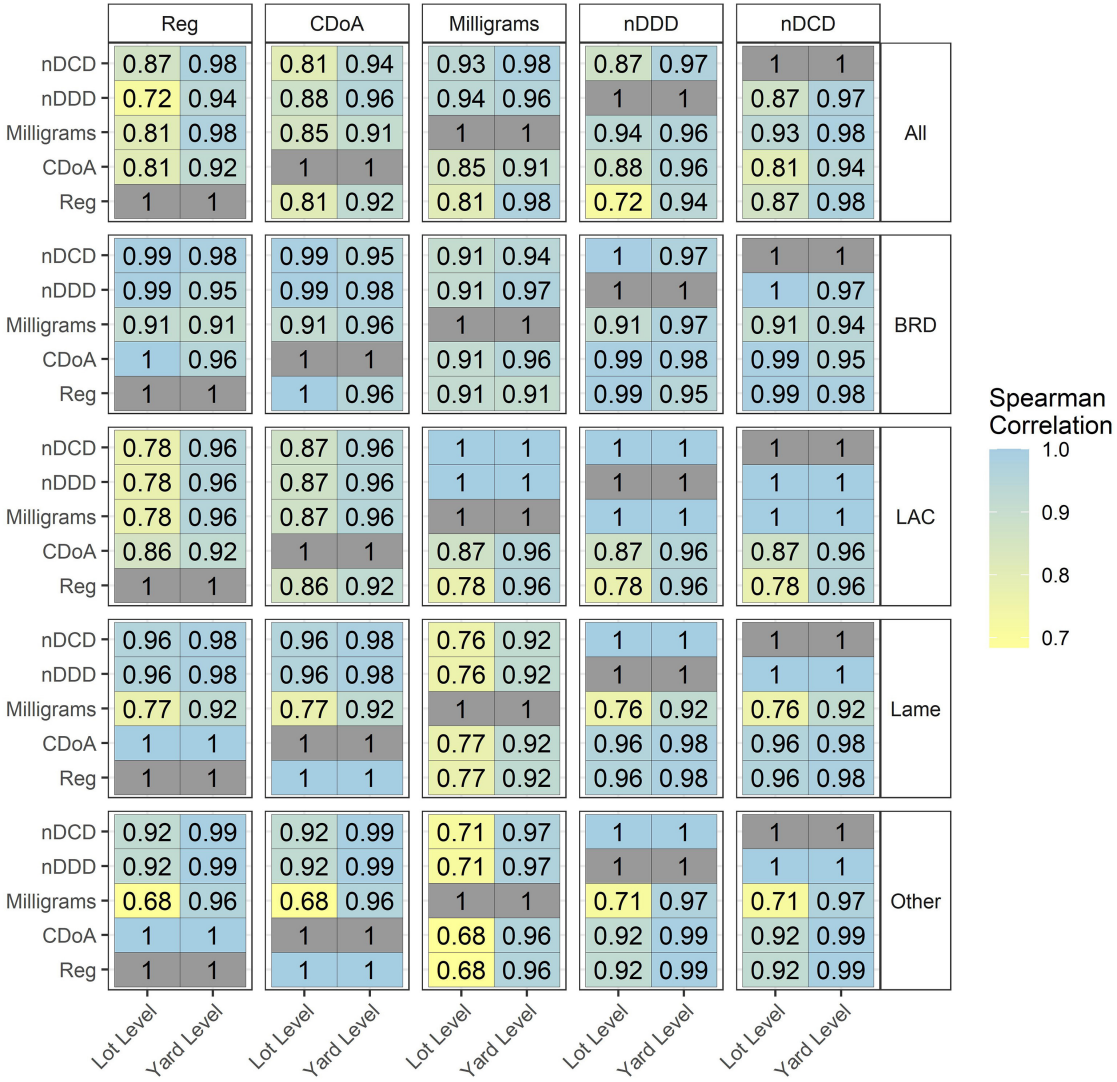
Correlation of 5 numerators within use indication at the lot and feedyard (yard) levels.

The correlations between 5 different numerators are presented in Figure 5, representing data from 2018 and 2019 combined. The indication labeled as “All” includes all the use indications where the numerators being correlated consist of all drug products used for all indications summed either within each lot (lot level) or across all lots within a feedyard (feedyard level). Other categories of use represent only that indication, consisting of BRD, LAC, Lame, and Other. In all cases, correlations are numerically greater at the feedyard level as compared to the lot level. The lowest correlations observed at the feedyard and lot levels were 0.91 and 0.68, respectively. The lowest correlations are observed for two relationships, (1) between mg and all other numerators within all use categories, and (2) between Reg and nDDD, nDCD, CDoA, or mg within LAC and “All” categories.

#### Denominators

**Figure 6:**
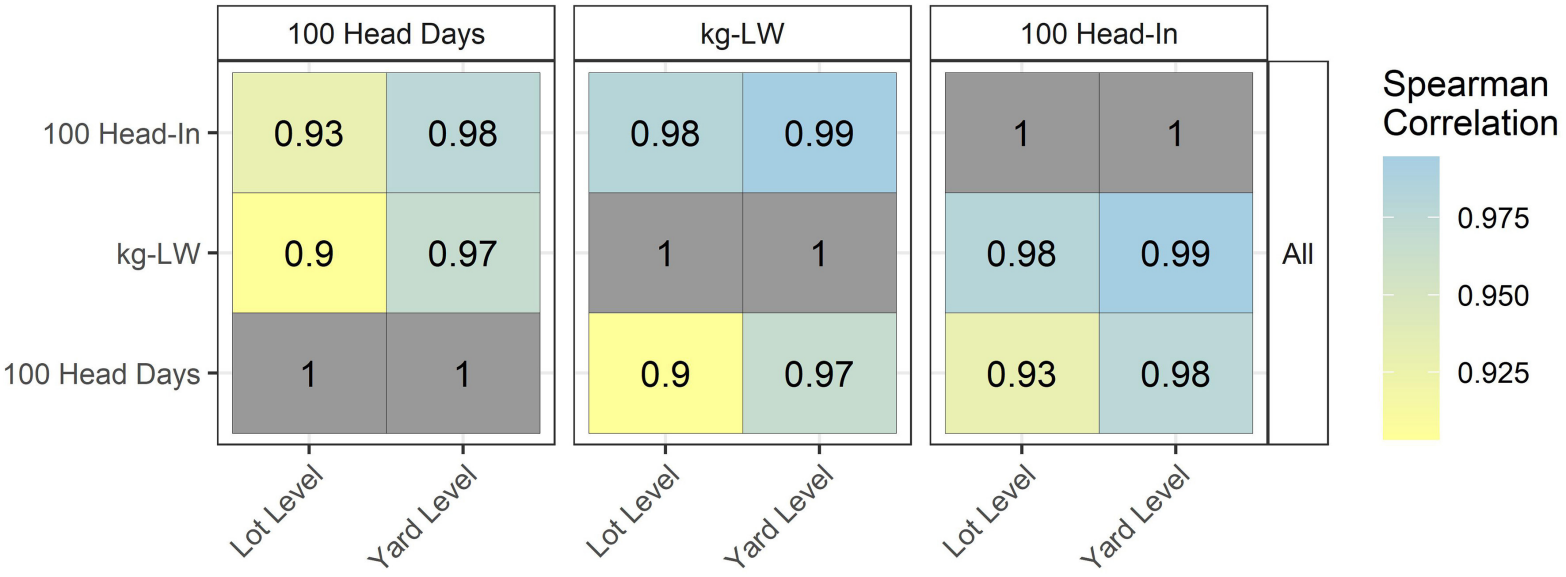
Correlation of 3 denominators at the lot and feedyard (yard) levels for all use categories combined.

The correlation of the denominators' 100 head days', kg-LW sold, and ‘100 head-in' are presented for all use categories combined in Figure 6. The correlations are high, with the lowest observed value of 0.90. As for numerators, the correlations are higher at the feedyard level.

### Correlations between 7 metrics by indication

**Figure 7:**
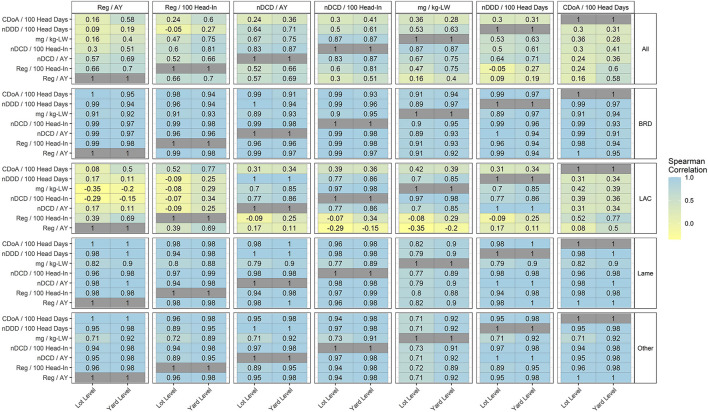
Correlations of 7 metrics for medically important antimicrobial use monitoring by use indication at the lot and feedyard (yard) levels.

Figure 7 reports correlations for metrics with 7 combinations of numerators and denominators. Note that the color scale encompasses a broader range of values in Figure 7 as compared to Figures 5, 6. As for numerators and denominators considered separately, the highest correlations are, with few exceptions, at the feedyard level.

Further consideration of contributors to differences in correlation between metrics may be informed by additional correlation tables in the Supplementary material. There, numerators are correlated across multiple denominators, and denominators are correlated across multiple numerators.

Correlations remain relatively high for most metrics within the use categories of BRD, Lame, and Other. Within LAC, the metrics nDDD/100 head days, nDCD/AY, nDCD/100 head in, and mg/kg-LW are relatively highly correlated, with the lowest observed correlation at the lot level of 0.7. In contrast, within LAC the metrics CDoA/100 head days, Reg/100 head in, and Reg/AY are less correlated, both with each other and with the other 4 metrics.

### Effect of metric choice on ranking of feedyards by antimicrobial use

**Figure 8:**
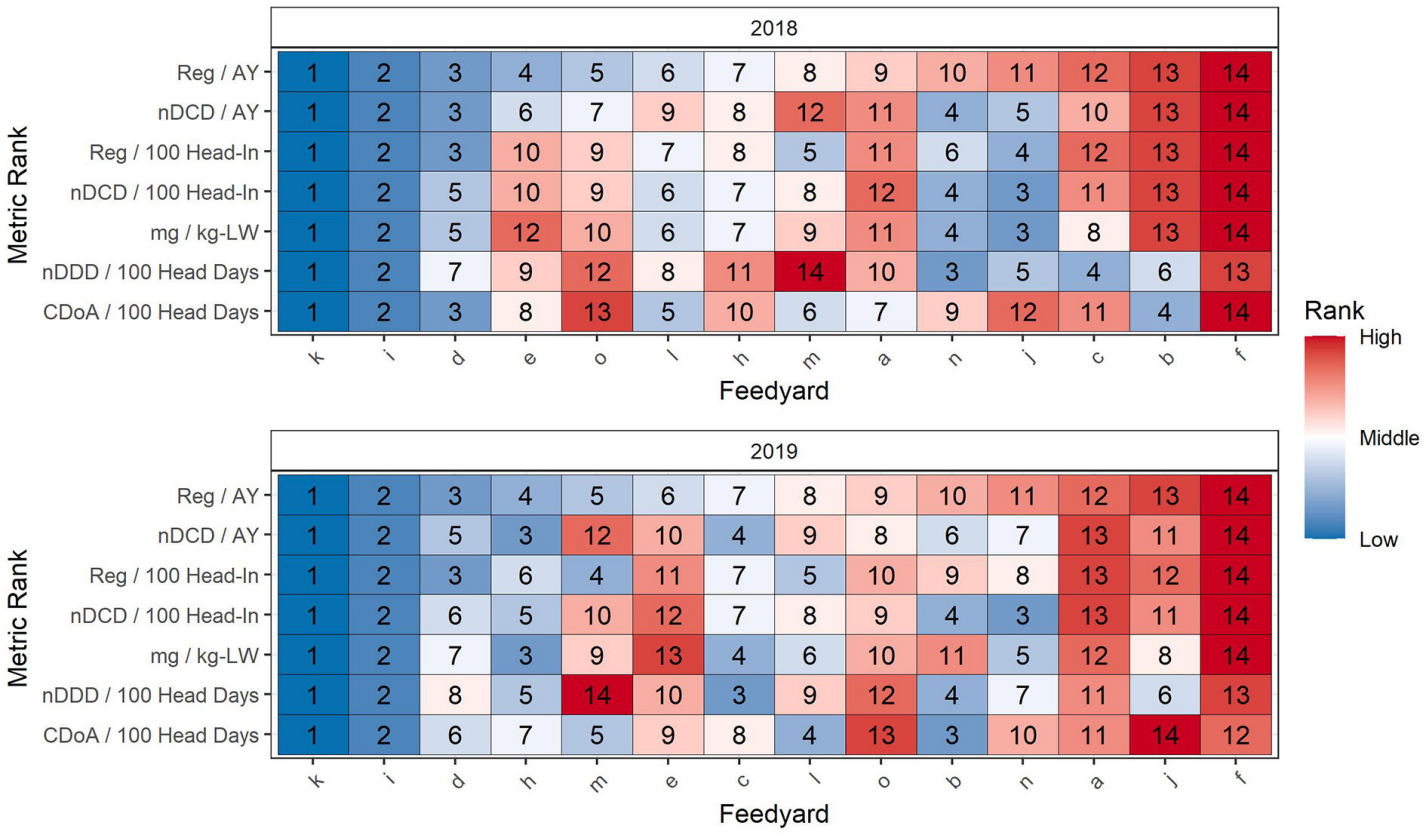
Comparison of feedyard antimicrobial use ranking by 7 metrics. Rank 1 is the lowest and rank 14 is the highest value. The feedyards are ranked on the X axis by their rank according to Reg/AY. Color coincides with numerical rank within each metric (row) as illustrated to the right of the figure.

Table 4 reports median and mean values at the feedyard level for the seven metrics used to rank feedyards. Ranking of feedyards relative to these 7 antimicrobial use metrics is reported in Figure 8, where feedyards are ranked by numerical order from 1 (lowest value) to 14 (highest value) with an associated color gradient of dark blue representing the lowest rank and red the highest rank. The feedyards, indicated by random alphabetical designations on the X axis, are ordered left to right according to their Reg/AY ranking in that year and each feedyard remains in that same column throughout the table for that year. For example, in Figure 8 data for 2018, Feedyard O is ranked 5^th^ by Reg/AY (the top row) and 13^th^ by CDoA/100 head days (bottom row).

**Table 4:** Median and mean values at the feedyard level for 7 metrics used in ranking feedyards.

**Metric**	**Year**	**Median**	**Mean**	**Std Dev**
CDoA / 100 Head Days	2018	89.2	74.3	33.7
	2019	77.0	72.4	34.7
nDDD / 100 Head Days	2018	80.1	76.0	33.1
	2019	79.1	71.6	35.6
mg / kg-LW	2018	25.7	24.7	13.3
	2019	22.6	26.1	18.8
nDCD / 100 Head-In	2018	139.0	128.9	58.4
	2019	115.2	128.5	73.5
REG / 100 Head-In	2018	131.4	126.0	57.0
	2019	119.2	122.0	67.5
nDCD / AY	2018	2.7	2.6	1.1
	2019	2.4	2.5	1.2
REG / AY	2018	2.8	2.6	1.2
	2019	2.4	2.3	1.2

Table 5 is a summary of differences related to which feedyards are included in the 4 lowest (28.6%) and 4 highest (28.6%) feedyards when feedyard rankings for the 7 antimicrobial use metrics in Figure 8 are compared. Comparisons are made for each study year within (1) metrics directly derived from the data (those with mg, Reg, or CDoA in the numerator), (2) within calculated metrics (those with nDDD or nDCD in the numerator), and (3) between directly derived and calculated metrics. For example, when comparing metrics directly derived from the data in Table 5 using REG/AY as the base ranking for comparison, there will be 1 different feedyard in the lowest 4 ranked feedyards and 1 different feedyard in the highest 4 ranked feedyards when using Reg/100 head-in as compared to Reg/AY as the metric for ranking. The feedyards which have changed may be determined by comparing the rankings for Reg/AY and Reg/100 head-in in Figure 8. Note in Figure 8 that within the bottom 4 ranking positions across both years and all metrics there are 2 outlier feedyards which are constant (k,i) in the bottom 4 positions. Within the highest ranked 4 feedyards, there is one feedyard which is consistently in the highest 4 (f).

**Table 5:** Differences in feedyards ranked in the lowest or highest 4 feedyards as compared to the designated base metric.

			**Number of feedyards differing from the base ranking**			
			**2018**		**2019**	
Comparison type	Base	Comparator	Lowest 4 (28.6%)	Highest 4 (28.6%)	Lowest 4 (28.6%)	Highest 4 (28.6%)
Comparison within metrics directly derived from the data	REG/AY	REG/100 head-in	1	1	1	1
		mg/kg-LW	2	2	1	2
		CDoA/100 head days	1	1	2	1
	mg/kg-LW	REG/100 head-in	1	1	2	1
		CDoA/100 head days	2	3	2	2
	REG/100 head-in	CDoA/100 head days	2	2	2	1
Comparison within calculated metrics	nDCD/AY	nDCD/100 head-in	1	1	2	1
		nDDD/100 head days	1	2	1	1
	nDCD/100 head-in	nDDD/100 head days	1	3	1	2
Comparison between metrics directly derived from the data and calculated metrics	REG/AY	nDCD/AY	1	2	1	1
		nDCD/100 head-in	2	1	2	1
		nDDD/100 head days	2	3	2	2
	REG/100 head-in	nDCD/AY	1	1	2	1
		nDCD/100 head-in	1	0	2	0
		nDDD/100 head days	2	3	2	2
	mg/kg-LW	nDCD/AY	1	1	0	2
		nDCD/100 head-in	0	1	2	1
		nDDD/100 head days	1	3	1	2
	CDoA/100 head days	nDCD/AY	1	3	2	1
		nDCD/100 head-in	2	2	1	1
		nDDD/100 head days	2	2	1	1

Within Table 5, with one exception in each column there is always at least one feedyard that moves out of either the 4 highest or 4 lowest feedyards in each of the 19 comparisons between the 7 metrics presented in Figure 8, and in many cases 2 feedyards. Within the lowest 4 ranking positions for 2018, in addition to the two consistently lowest ranking feedyards there are 6 other feedyards that in at least one metric are ranked in the lowest 4. This number for 2019 was 7. In the highest ranked group of 4 for 2018, outside of the one consistently highest ranked feedyard there are 8 other feedyards which within at least one metric are ranked in the highest 4 feedyards. For 2019 this number is 7. Interestingly, in 2018, 4 of the 14 feedyards (e, j, c, b) appear in both the lowest and highest 4 ranked feedyards within different metrics. This number in 2019 was 3, although 2 of the feedyards are different from 2018 (b, m, n).

**Figure 9:**
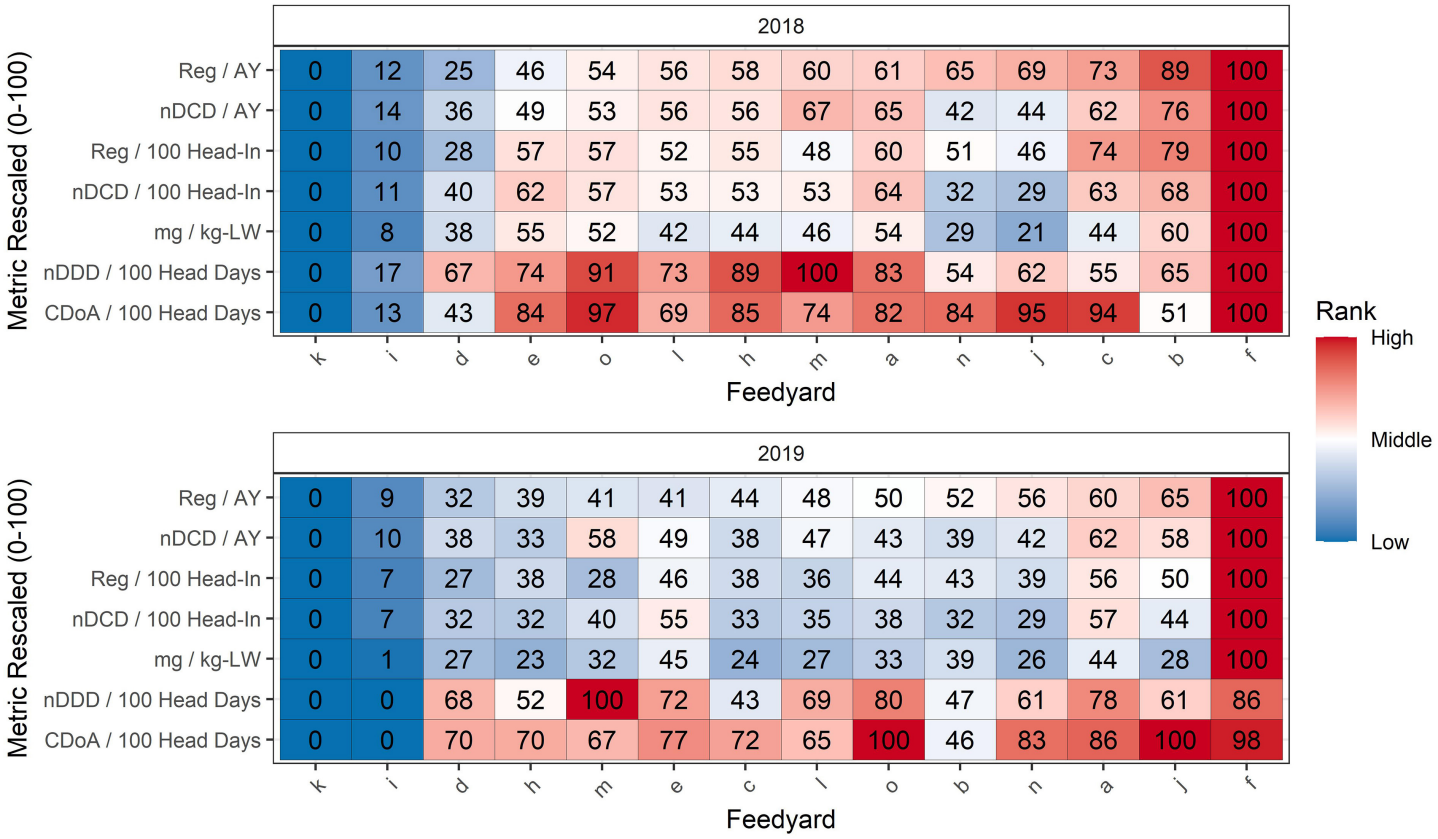
Comparison of feedyard antimicrobial use ranking by 7 metrics rescaled to be expressed as proportional ranks. The feedyards are ranked on the X axis by their rank according to Reg/AY. Color coincides with numerical rank within each metric (row) as illustrated to the right of the figure.

In Figure 9, the feedyards are ordered by their rank rescaled in proportion to their actual value in relation to the range of observed values scaled as 0 (lowest) to 100 (highest); this proportional ranking reflects both order and magnitude of difference from other feedyards. Color is again associated with the ranking, from dark blue for the lowest to red for the highest. The two lowest and the single highest ranked feedyards are consistent across all 7 metrics as they set substantially different outlier values for the data. Of the two lowest feedyards, one did not use tylosin in the feed for reduction in incidence of liver abscesses in either year, and the other used tylosin for only the first few months of 2018 before discontinuing the practice. Note that the proportional ranking within the feedyards in the middle are quite close as compared to their relationships with the consistent lowest and highest feedyards.

Table 6 presents the results of the Wilcoxon rank analysis between pairs of metrics. In both years of the study, there were three comparisons where the *P* value was not sufficient to reject the null hypothesis that the ranking was the same between the two metrics. Note that these are the comparison of Reg/AY to nDCD/AY, the comparison of Reg/100 head-in to nDCD/100 head-in, and the comparison of CDoA/100 head days to nDCD/100 head days. These 3 comparisons are placed next to each other in Figures 8, 9.

**Table 6:** Wilcoxon rank analysis of differences in feedyard ranking between different metrics (N = 14 feedyards, 19 metric comparisons).

**Metric 1**	**Metric 2**	**Year**	***P* value**
Reg/AY	nDCD/AY	2018	0.5416
		2019	0.5016
Reg/100 head-in	nDCD/100 head-in	2018	0.4631
		2019	0.3910
CDoA/100 head days	nDDD/100 head days	2018	0.4263
		2019	0.8552
All other metric ranking comparisons		2018	≤ 0.0006
as illustrated in Table 5		2019	≤ 0.0012

The 3 ranking comparisons in Table 6 with non-significant *P* values may also be evaluated in Table 5 and Figures 8, 9. Note that even when a difference in ranking is not demonstrated as being statistically significant, the metric used may still make a difference for individual feedyards in being considered within the lowest or highest antimicrobial users. When Reg/AY was compared to nDCD/AY in 2018 there was still one feedyard different in the bottom 4 rankings and 2 feedyards different in the highest 4 rankings. Similarly, for CDoA/100 head days as compared to nDDD/100 head days, 2 feedyards were different in both the lowest and highest ranked groups of 4 in 2018.

## Discussion

### Variation inherent in beef feedyards

Antimicrobial use reporting may be used to describe a combination of multiple food animal industries within a country, a food animal industry, different production entities within an industry (feedyards presented here), evaluation of specific groups of animals within each production entity (lots presented here), or antimicrobial use linked to each specific animal. In the data reported here, the individual animal granularity of injectable drug treatment data, and lot-level granularity of in-feed data, made it possible to start at the individual animal level and combine data up to the feedyard level.

The U.S. feedyard environment displays unique variation within and between feedyards which affects the relationship of different antimicrobial use metrics. Each lot has a varying period of time present in the feedyard as compared to others. The sum of days each animal in the lot was present in the feedyard is expressed as total head days. Head days may vary greatly by the type of animal being fed. Small Holstein steers may be fed for as long as a year to reach market weight while a heavy yearling steer may be fed as little as 120 days; 3 yearling steers would be fed in the same number of head days as one Holstein steer calf. Light beef calves are often fed 240–260 days depending on genetic potential, ration, time of year, market influences, and sex, with heifers typically finishing at a lighter weight than steers. An example of the effect of head days (also referred to as days on feed) on Reg/AY and mg/kg-LW for tylosin fed to reduce the incidence of liver abscesses has been published (8).

The portion of the feeding period at risk for the indications reported here vary by indication. The Liver Abscess Control (LAC) use indication consists entirely of tylosin, which may be fed starting in the first ration or may not be started until the final ration when the highest energy content is reached, which may occur after approximately 20–30 days in the feedyard depending on the feeding program. Tylosin may be fed continuously until the cattle are sold or may be discontinued the last 30–45 days of the feeding period.

Lameness may occur anytime during the feeding period. In a review of 2,532 cases of feedyard lameness, Terrell, et al. (10) reported that the average days in the feedyard at the time of diagnoses was 57 ± 48 days when considering 9 types of lameness.

When considering BRD, the common concept is that this disease primarily occurs early in the feeding period. However, that concept may best fit with the classification of “high-risk” calves which are typically composed of groups of calves originating from multiple sources and transported long distances. In a comparison of high-performing and high-risk calves, Theurer et al. (11) found that by 45 days on feed the high-risk calves had demonstrated 67.2% of the total BRD cases observed during the feeding period while the high-performing calves had only displayed 33.7% of the BRD cases which would be diagnosed during the entire feeding period. The incidence of BRD also varies between steers and heifers, and is expected to be lower with heavier weight cattle entering the feedyard, although these heavier cattle may display a higher proportion of their BRD mortality later in the feeding period (12). A common practice is to view feedyard benchmarking data for disease or performance by in-weight categories and sex. Timing of feedlot placement may also be considered.

The “other” indication for antimicrobial treatment consists of a variety of infectious diseases, such as central nervous system disease due to *Histophilus somni* or *Listeria monocytogenes*, infectious bovine keratoconjunctivitis (pinkeye) due to *Moraxella bovis*, or cellulitis. The incidence of these diseases is comparatively low and sporadic, and therefore they are combined as a single indication in the data reported here.

Feedyard populations also vary by time of year. The peak feedyard placements in the United States typically occur in August through October when the majority of calves are weaned as a result of the majority of beef cows calving in the spring. Heavier cattle which have been on pasture also enter the feedyards at this time due to grass going into winter dormancy in a large portion of North America. Market influences, cow inventories, availability of small grain winter pasture on the high plains, drought, and available feedyard capacity all heavily influence the timing of placements from year to year.

### The use of DDD and DCD in these calculations

It could be argued that the DDD and DCD values calculated for this study could be presented as used daily doses (UDD) and used course doses (UCD) since the calculations came directly from the population being evaluated. However, DDD and DCD are used in this report due to a single value being applied to multiple populations (individual feedyards) for which their doses may vary, and the values calculated for all feedyards combined may not be representative of an individual feedyard.

The result of these two approaches is that each lot and each feedyard had mg, Reg, and CDoA numerator values for each drug product determined directly from the records. Each lot and each feedyard also had a nDDD value calculated for each drug product from the feedyard or lot specific total mg and the study defined DDD values, as well as a nDCD value calculated for each drug product from the feedyard or lot specific mg and study defined DCD values. A total mg, Reg, CDoA, nDDD, or nDCD value for a lot or feedyard was calculated by adding this value for all drug products recorded for that lot or feedyard. The feedyard level values for each numerator are sums of lot values, not means of lot values.

Although DDD and DCD values are typically expressed as mg/kg, then applied to an estimated average weight at the time of treatment, the kg denominator was not necessary for the calculations reported here. By using the total values for mg, Reg, and CDoA from the study population for calculation of DDD (mg/CDoA) and DCD (mg/Reg), the mean and distribution of the weights at the time of treatment were the same for all methods of calculating numerators. In other words, instead of estimating the mean weight at the time of treatment for the population and then applying a mg/kg estimate for DDD or DCD to this weight estimate, the DDD and DCD were calculated directly from the population records, giving the best possible match of the DDD and DCD values to the population.

### Using nDDD to estimate CDoA and nDCD to estimate Reg

This study represents the best-case scenario for comparing the relationship of nDDD to CDoA, and nDCD to Reg within U.S. feedyards. The mean kg bodyweight at the time of treatment for DDD and DCD values were exactly those of the population from which CDoA and Reg were calculated, respectively. An estimated number of days of therapy (DOT) associated with single injection antimicrobial products was not assigned in this study and assigning the same value to both CDoA and in estimating DDD would not have altered the relationship between these two metrics. However, it would have changed the proportional effect of single injection drugs on the overall values for CDoA and DDD in relation to in-feed routes of administration by increasing the number of CDoA and DDD related to each single injection product.

Despite the ideal conditions in this study, significant error is still possible when using nDDD and nDCD values to rank lots or feedyards by days of antimicrobial exposure or number of animals exposed, respectively. Using DDD to estimate CDoA resulted in a >25% over or under estimation in 50% of feedyards in 2018, 21.4% of the feedyards in 2019, and 49.2% of the lots across both years. Using DCD to estimate the number of animals exposed to an antimicrobial regimen resulted in 21.4% of the feedyards and 46.4% of the lots differing from actual regimen counts by >25%. These errors would be compounded when the kg weight at the time of treatment differed between the DDD and DCD estimates and the mean weight of the population to which these values are applied.

### Correlations between numerators and between denominators

Correlation coefficients for numerators displayed a minimum of 0.91 at the feedyard level and 0.68 at the lot level. The higher correlations at the feedyard level reflect the decrease in variation resulting from expressing total values for each feedyard. The lowest numerator correlations were seen within LAC due to the wide variation in mg/Reg and CDoA/Reg for LAC, previously reported as mean ± SD values of 12,734 ± 5,990 mg and 154 ± 58 days, respectively (7). This previously reported mean mg/Reg value (12,734 mg/Reg) differs from the value reported here (12,543 mg/Reg) for the same data set by 1.5% due to the decision to eliminate several extreme outliers included in the previously reported analysis. This decision was made due to the concern that these outliers would inappropriately skew the relationships between metrics. This same approach also resulted in a 0.8% difference in chlortetracycline in-feed mg/Reg values between the two papers.

While all use categories varied by mg/Reg within and between products, LAC added the large variation in CDoA/Reg also. The effect of LAC variation was carried through to the “All” use indication, with the effect dominating the other use categories due to the use of tylosin for LAC consisting of 71.4% of regimens. Correlations between denominators were uniformly high. The correlation between 100 head-in and kg-LW sold was the lowest at 0.90 and 0.97 at the lot and feedyard levels, respectively. These results suggest that, within the metrics evaluated here, selection of a numerator has more effect on apparent antimicrobial use than does any one of these tested denominators. This statement should not be interpreted as dismissing the importance of accuracy and relevancy of denominators used for antimicrobial use evaluation, especially when used for comparisons.

### Correlations between 7 defined metrics

While separate evaluation of correlation within numerators and within denominators gives the initial impression that the resulting metrics should also be highly correlated, this impression is not supported by the correlations of combined numerator and denominator metrics presented in Table 6.

Correlations between metrics are relatively high within the BRD, Lame and Other use categories due to dominance by single injection antimicrobials with a similar distribution of use across all feedyards. For example, of the 25% of total regimens not representing in-feed administration, tulathromycin accounts for 7.9%, ceftiofur crystalline free acid accounts for 4.8%, and oxytetracycline products account for 4.8% of total regimens.

The highest variation and lowest values for correlation are for LAC. The relatively high correlation of the metrics nDDD/100 head days, nDCD/AY, nDCD/100 head in, and mg/kg-LW within LAC might be expected as nDDD and nDCD values are calculated from mg values for each product, with fixed relationships between DDD and DCD values for each product. Also, the denominators expressed as head-in, 100 head days, and animal years are highly correlated; animal years and 100 head-days are calculated from the same base value of total head days, which is highly correlated with head-in.

In contrast, the relatively poor correlation of the 4 metrics discussed above with the other 3 metrics (CDoA/100 head days, Reg/100 head-in, and Reg/AY) within LAC may be attributed to the low correlation between DDD and DCD values with CDoA and Reg, respectively, within the LAC indication. The lower correlation between Reg/AY and Reg/100 head-in (0.38) illustrates the complicated interaction of numerators and denominators beyond the correlation of each; in this case, the same numerator is placed over highly correlated denominators. The reduced correlation between Reg/AY and Reg/100 head-in is due to there being minimal variation in Reg/100 head-in and large variation in Reg/AY due to different lengths of regimens related to different feeding periods.

The “All” use indication displays reduced correlation values as compared to BRD, Lame, and Other due to the inclusion of LAC antimicrobial use into this all-inclusive metric. In addition, note that the correlation between nDDD/100 head days and nDCD/AY is 1 in the specific use categories (BRD, LAC, Lame, and Other) but is reduced in “All”. This is due to the distributions being highly correlated for each specific use indication but having very different shapes of the distributions. When combined into the “All” use indication, the combination of different distributions results in the overall correlation being much less than when each use indication is evaluated separately.

### Effects of metric selection on feedyard antimicrobial use ranking

Figures 8, 9 use the same metrics as correlated in Table 6 and rank feedyards either in a simple numerical order or by a scaled rank. Table 5 summarizes ranking differences encountered in multiple metric comparisons and illustrates that variation in ranking is consistent across all metric comparisons. The finding that 4 of the 14 feedyards (4 out of the 11 non-outliers) were represented in both the highest and lowest use groups by at least one metric in 2018 illustrates the effect imparted on antimicrobial use reporting by the selection of a metric or metrics for comparison. The overall conclusion for numerical ranks is that the outliers are consistent across all metrics and some of the middle feedyards are affected much more in ranking by different metrics as compared to other feedyards.

The ranks are rescaled in Figure 9 so that each value represents the proportional ranking along a scale from 0 for the lowest value to 100 for the highest value, illustrating the tighter grouping of the middle 11 feedyards compared to the outliers, suggesting that this tight grouping in the middle is responsible for the larger effect of metrics on ranking in the middle of the ranks as opposed to the ends. The proportional values support the observation that identifying the highest-use outliers is possible with essentially any metric, but that the picture of ranking within the main body of antimicrobial use is heavily influenced by the metric selected. The reader is left to consider the additive effects of different estimated mean weights at treatment and different DDD and DCD values used in calculating different metrics, especially when they are used to compare different countries.

The Wilcoxon rank test failed to demonstrate a significant difference in ranking between the three metric pairs which display the most similarity, while showing highly significant differences for all other metric comparisons. While this may be true as far as overall ranking, when considering the lowest or highest use groups, each of the pairs within these comparisons still resulted in different feedyards being considered as being in the lowest or highest use groups. This supports the contention that focusing on outliers may be an effective first-phase approach to antimicrobial stewardship, but soon thereafter the ranking of closely clustered production entities becomes essentially arbitrary based on the metric selected.

### Other studies comparing antimicrobial use metrics

#### Comparison of antimicrobial use metrics in North American beef feedyards

Brault et al. (13) evaluated different methods of calculating antimicrobial use metrics for individual animal administration of injectable antimicrobials in Canadian feedyards. In evaluating the use of estimated vs. actual weights of cattle at the time of exposure, they found that in their dataset the use of mean weights at the time of antimicrobial exposure underestimated the number of Animal Daily Doses (nADD) by 7.3%. This effect was even more pronounced for some of the antimicrobial classes, notably underestimation of macrolide use by 23.2% and beta-lactam use by 43.1%. The mean weight at the time of exposure was 336 ± 98 kg, with mean weights at the time of exposure for macrolides and beta-lactams of 267 kg and 484 kg, respectively. The issue of differing estimated weights at the time of exposure has also been highlighted in veal calves, with estimations of 172, 86, 70, and 140 kg being used in calculations for the Netherlands, Denmark, France, and the ESVAC project, respectively (2). Emphasizing the need for consistent DDD and DCD values is of questionable value when both the doses and the estimated weights at time of treatment may vary substantially between jurisdictions.

Brault et al. (13) also evaluated the use of dose-based and weight-based metrics in feedyards, concluding it can “clearly be seen that mg as a measurement of antimicrobial use confuses interpretation when different classes of antimicrobial drugs are used at disparate levels in the two populations compared”. These researchers also concluded that different duration of effect estimates applied to single injection antimicrobials and different biomass estimates have large effects on the final estimates.

#### Other studies comparing antimicrobial use metrics in food animals

In a 2012 analysis of data for Denmark and the Netherlands, Bondt et al. (14) compared computation of use from national sales data as compared to detailed census data obtained from individual producers. The authors concluded that “although the computed antimicrobial exposure would seem to be a reasonable estimation of the exposure for all animals as a whole, it differs significantly from the measured exposure for most species”. In addition, they stated “The conclusion is that simple country comparisons, based on total sales figures, entail the risk of serious misinterpretations, especially if expressed in mg per kg. The use of more precise model calculations for making such comparisons, taking into account differences in dosages and in farm animal demographics, only slightly reduces this risk”.

A study ranking 67 Irish pig farms according to antimicrobial use reported by 7 different metrics found that 15 farms were above the arbitrary top 25% action level (highest 17 farms) for at least 6 of the 7 metrics (15). Ten farms were above the action level for all 7 metrics. However, a reported lowest Spearmans correlation coefficient of 0.94 is not that surprising when all of the numerators evaluated were calculated from the weight of drugs used, and weights of pigs vary less than cattle. The main source of variation between farms from the drug weight-based reporting would be in the relative amounts of different antimicrobials being used and how that affected the proportion of different conversion factors from weight of drug to DDD values. It is important to recognize that many of these different metrics are not different measurements, they are different ways of expressing the same weight of drug used; they are different calculation methods, not measures.

A recent study evaluated mass-based and dose-based metrics (calculated from drug mass records using an animal daily dose and average weight at time of treatment) for antimicrobial use on dairy farms (16). The investigators reported that the two metrics gave different impressions of the primary indications for antimicrobial use as well as the primary drugs used. For example, a dose-based approach indicated that intramammary administration was 78% of total animal daily doses (ADD) while a mass based approach indicated that only 24.1% of total AMU was intramammary. The ADD approach indicated ceftiofur was the predominant antimicrobial used, at 53% of total use, while the mass-based approach indicated the predominant antimicrobial used in cows was ampicillin, at 33 % of use. In this study, both the predominant route and predominant drug were different depending on the metric used.

There are other reports in swine, turkeys, and broiler chickens evaluating influences such as substituting different numerators or utilizing actual used doses vs. recommended doses (17, 18). These studies support the central theme that the selection of the antimicrobial use metric matters, and applying the same calculation methods across disparate populations does not assure an accurate comparison of antimicrobial use.

## Conclusion

The analysis reported here adds to the body of literature reporting substantial effects of metric choice on the conclusions drawn from comparing antimicrobial use across multiple production sites. Of the 14 feedyards in this study, 2 feedyards were consistently in the lowest ranked group of 4 feedyards and one feedyard was consistently in the highest ranked group of 4. Six other feedyards were included in either the lowest or highest ranked groups of four within at least one metric in one of the study years. Contributing to this variation is the dominant use of an in-feed antimicrobial for liver abscess control with an extended and variable regimen duration and large differences in head days for individual lots. The choice of metric matters in the ranking of individual feedyards, especially within the more tightly grouped feedyards in the middle of the values. There may also be substantial differences in nDDD vs. CDoA and nDCD vs. Reg for individual feedyards and lots.

Whether the selected antimicrobial use metrics are being used for policy decisions, marketing competition, or antimicrobial stewardship evaluations, an understanding of the nuances of each metric in each population is a requirement for rational and equitable use of the information. After the initial antimicrobial use outliers are corrected or removed, we must be careful to not fulfill the prediction of Marilyn Strathern, who cautioned “When a measure becomes a target, it ceases to become a good measure” (19).

## Data availability statement

The datasets presented in this article are not available because a condition of participation by the feedyards contributing data was that their identity and the raw data were to remain confidential. Therefore, the raw data are not available. Requests to access the datasets should be directed to mapley@vet.k-state.edu.

## Author contributions

MA recruited the feedyards, coordinated data collection, and wrote the manuscript drafts. NS and DA conducted the data analysis, wrote the R code, and contributed to the paper content. BL and RS contributed to the project direction from conception, participated in determination of analytical methods and quality assurance, evaluated clarity of data presentation, and commented on manuscript drafts. All authors contributed to the article and approved the submitted version.
