# Supplementary material for: The association of multiple metrics for evaluating antimicrobial use in U.S. beef feedyards
Source: Front Vet Sci. 2023 Jan 4;9:1056476. doi: 10.3389/fvets.2022.1056476 (PMC9848654; doi:10.3389/fvets.2022.1056476)

The association of multiple metrics used for evaluating antimicrobial use in U.S. beef feedyards.

Supplemental information

This supplemental document provides a table of abbreviations and calculations, and additional correlation tables for multiple numerator-denominator metric combinations.

Contents

1 Table of abbreviations and selected calculations 2
2 Correlation of a numerator over multiple denominators 3
3 Correlation of multiple numerators over the same denominator 6

Tables

Table 1 Selected abbreviations and calculations 2

Figures

Figure 1 Correlation of regimen (Reg) over four denominators within indication for use at the lot and feedyard (yard) levels. 3
Figure 2 Correlation of milligrams (mg) over four denominators within indication for use at the lot and feedyard (yard) levels. 4
Figure 3 Correlation of calendar days of administration (CDoA) over four denominators within indication for use at the lot and feedyard (yard) levels. 5
Figure 4 Correlation of 5 numerators over the denominator "animal year" (AY) within indication for use at the lot and feedyard (yard) levels. 6
Figure 5 Correlation of 5 numerators over the denominator "100 head in" within indication for use at the lot and feedyard (yard) levels. 7
Figure 6 Correlation of 5 numerators over the denominator "kilograms liveweight sold" (kg-LW) within indication for use at the lot and feedyard (yard) levels. 8

1 Table of abbreviations and selected calculations

Table 1 Selected abbreviations and calculations

AY	Animal Year - The total number of days animals were in the feedyard, summed for either a lot, feedyard, or the entire study population, and then divided by 365 to express the value as years.
BRD	Bovine Respiratory Disease
CDoA	Calendar Days of Administration - The number of days a drug was administered during a regimen.
DCD	Defined Course Dose - Calculated here across the entire study population by dividing the total milligrams of drug administered for a use indication by the total recorded regimens for that drug and use indication. Defined course doses are defined at the product level.
nDCD	The number of Defined Course Doses determined for a population, calculated by dividing total mg of a product for a population by the defined course dose.
DDD	Defined Daily Dose - Calculated here across the entire study population by dividing the total milligrams of drug administered for a use indication by the total recorded calendar days of administration for that drug and use indication. Defined daily doses are defined at the product level.
nDDD	The number of Defined Daily Doses determined for a population, calculated by dividing mg for a population by the defined daily dose.
Kg-LW	The kilograms of animals sold expressed as weight measured when leaving the feedyard.
LAC	Liver Abscess Control - While this term is used to describe the use in this paper, the label indication for tylosin is for reduction of the incidence of liver abscesses.
Lame	All antimicrobial uses attributed to any cause of lameness.
Other	All antimicrobial uses attributed to any cause not attributed to bovine respiratory disease, liver abscess control, or lameness.
Reg	Regimen - A single antimicrobial administration or a series of consecutive antimicrobial administrations associated with one antimicrobial product, one animal, and a use indication where the time gap between administrations belonging to the same regimen is never greater than 2 days.

2 Correlation of a numerator over multiple denominators

Figures 1, 2 and 3 report the correlations of the numerators Reg, mg, and CDoA, respectively, expressed over each of 3 denominators. Correlations within the Bovine Respiratory Disease (BRD), Lameness (Lame), and "other" indications for use remain 0.93 or above for all numerator – denominator combinations. The lowest correlations are consistently displayed within the Liver Abscess Control (LAC) indication, with Reg/kg-LW sold and Reg/AY displaying the lowest observed correlation. The effect of the LAC indication for use is apparent when all use indications are combined in the "All" use indication.

Figure 1 Correlation of regimen (Reg) over four denominators within indication for use at the lot and feedyard (yard) levels.

Indications for use are indicated to the right.

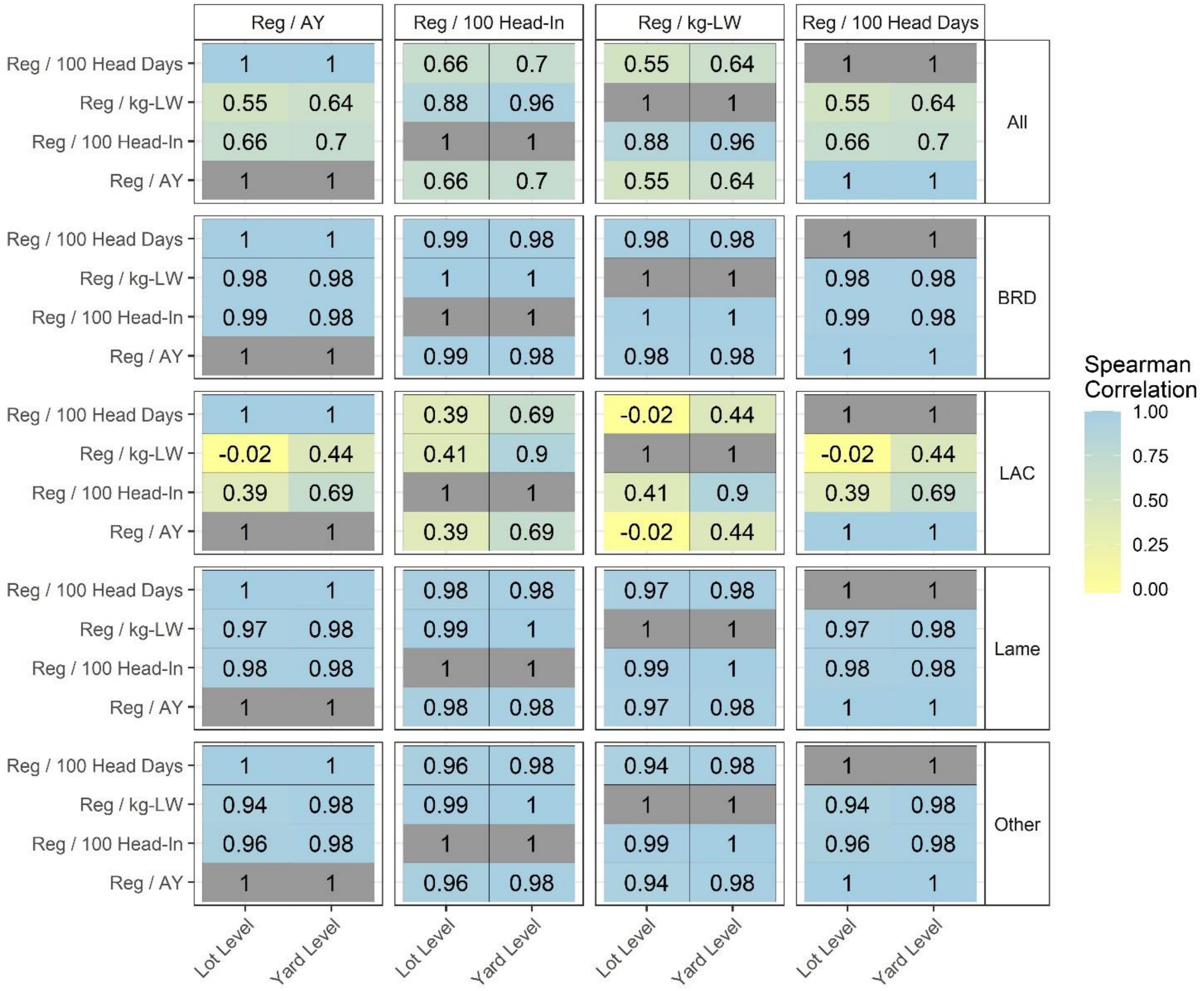

Figure 2 Correlation of milligrams (mg) over four denominators within indication for use at the lot and feedyard (yard) levels.

Indications for use are indicated to the right.

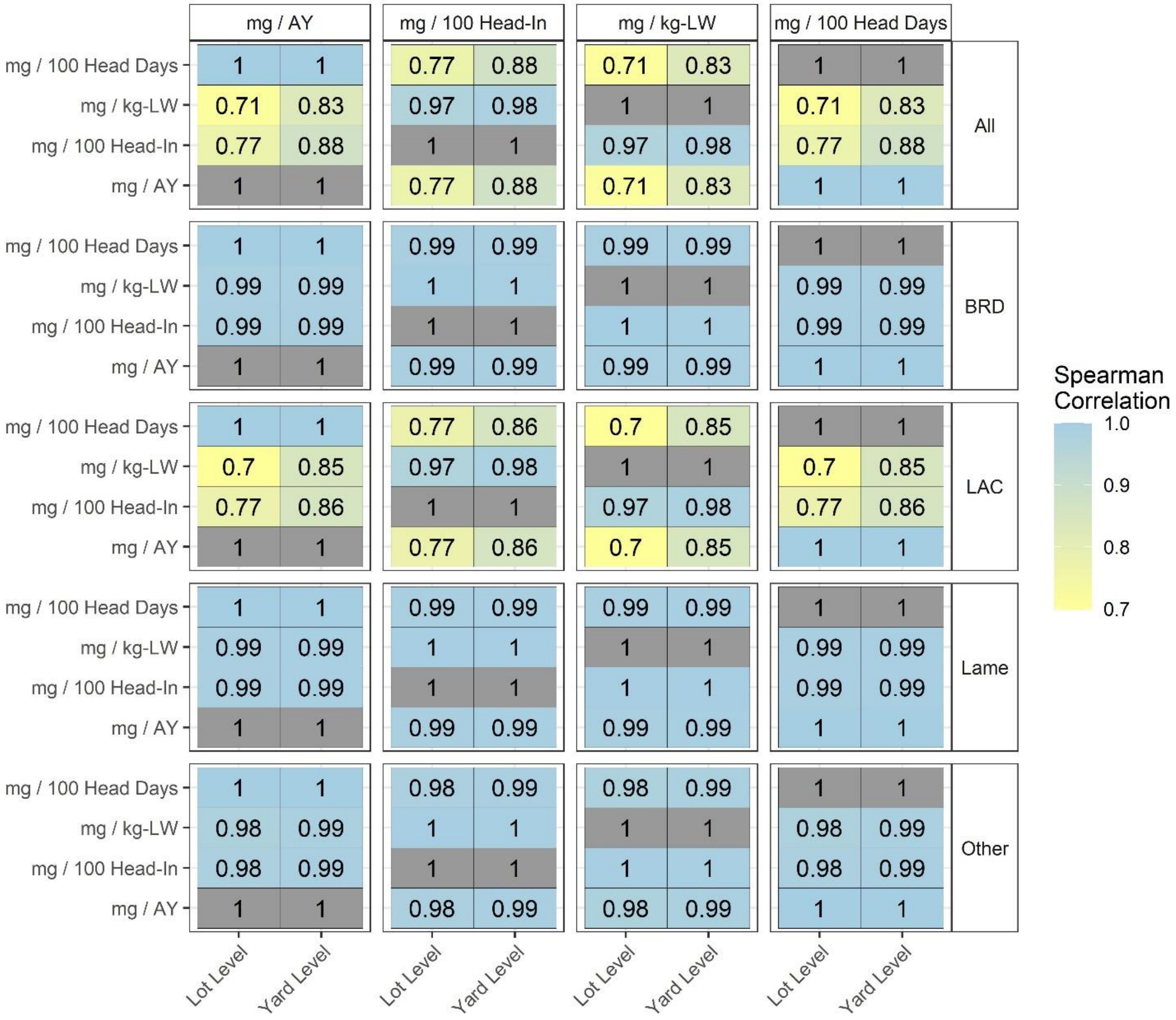

Figure 3 Correlation of calendar days of administration (CDoA) over four denominators within indication for use at the lot and feedyard (yard) levels.

Indications for use are indicated to the right.

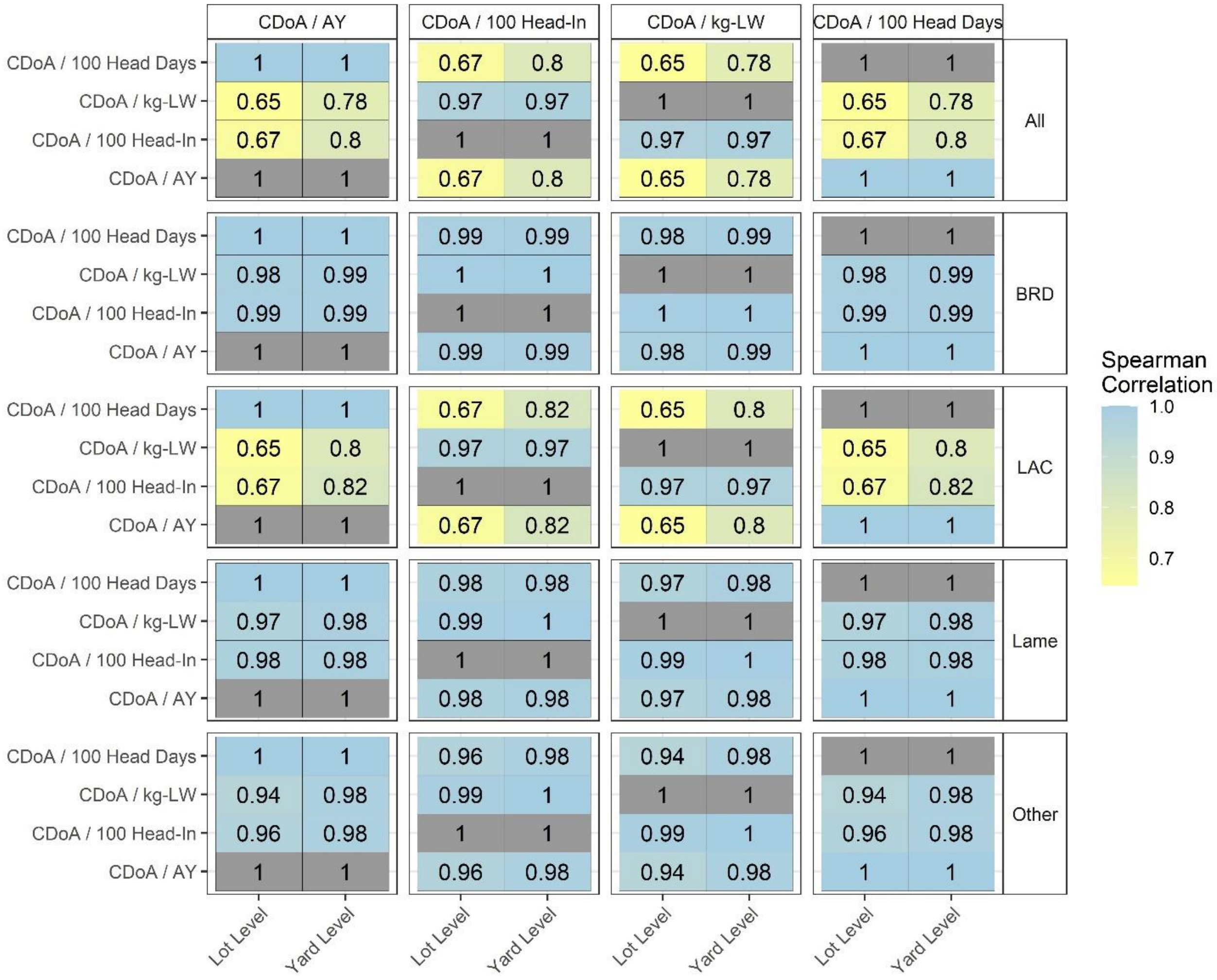

3 Correlation of multiple numerators over the same denominator

The correlations of multiple numerators over the denominators AY, head-in, and kg-LW sold are presented in Figures 4, 5, and 6, respectively. Correlations are presented within indication at the lot and feedyard levels. The lowest correlations for a numerator are observed with mg, and the lowest correlations within an indication are observed within Liver Abscess Control (LAC). The consistent trend of higher correlation values at the feedyard level is continued in Tables 4, 5, and 6.

Figure 4 Correlation of 5 numerators over the denominator "animal year" (AY) within indication for use at the lot and feedyard (yard) levels.

Indications for use are indicated to the right.

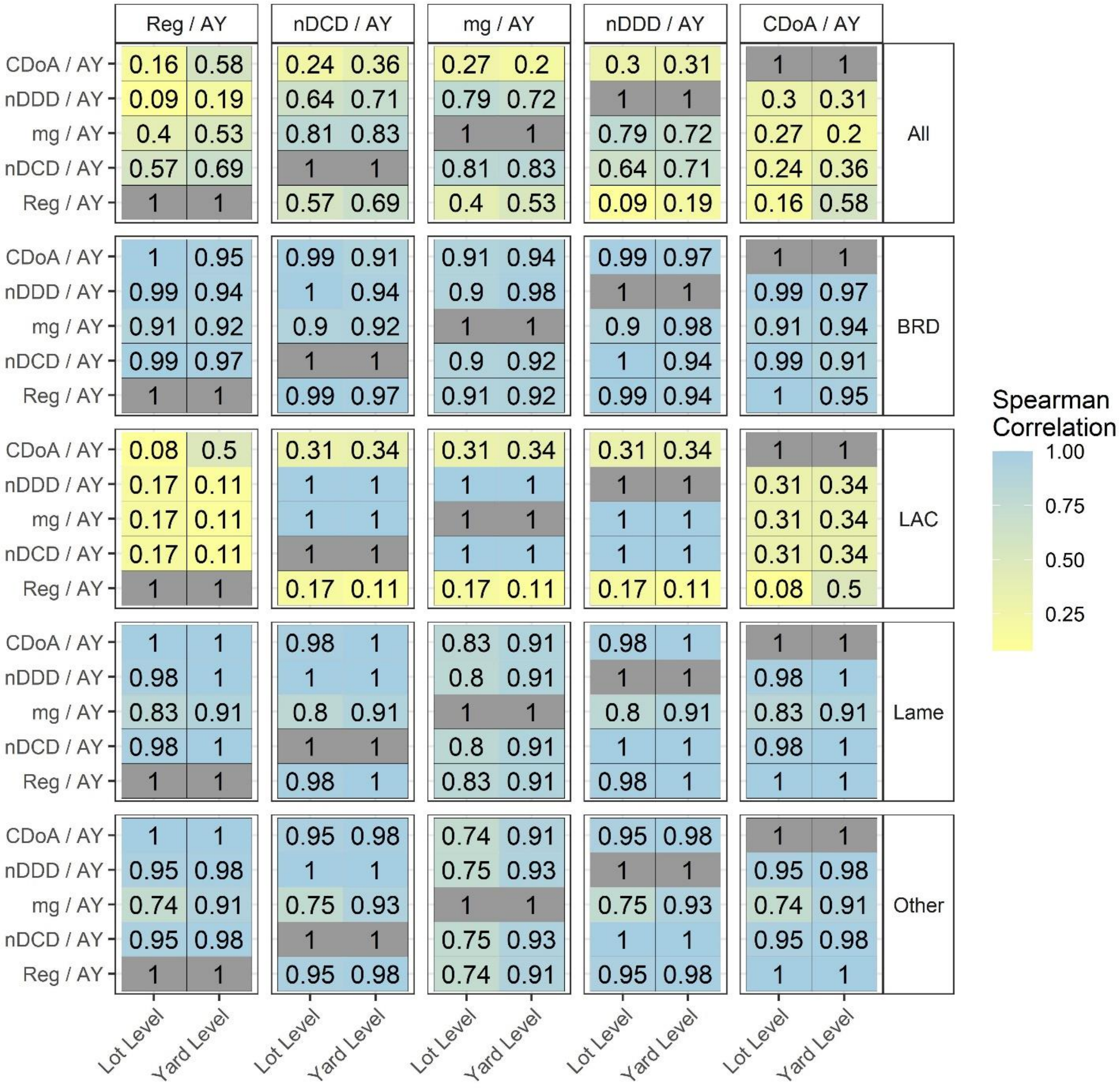

Figure 5 Correlation of 5 numerators over the denominator “100 head in” within indication for use at the lot and feedyard (yard) levels.

Indications for use are indicated to the right.

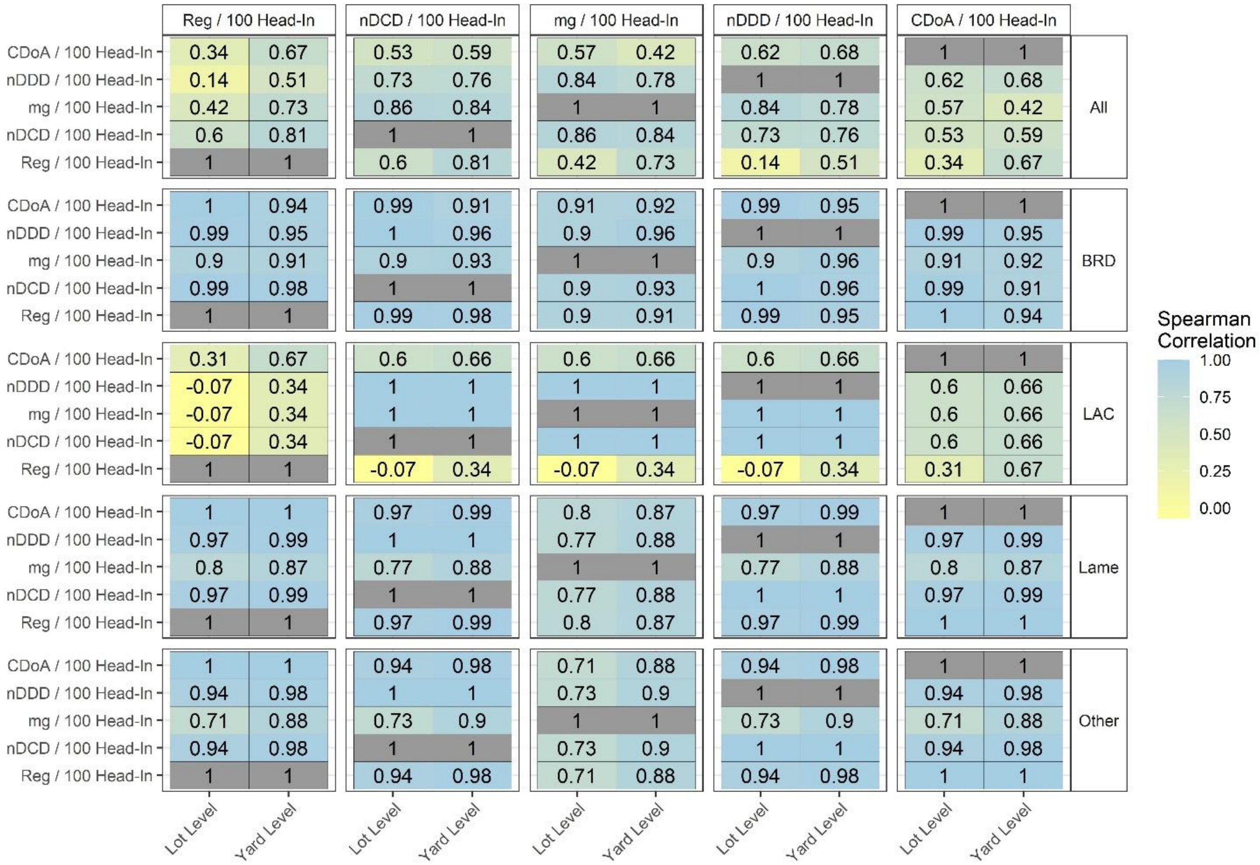

Figure 6 Correlation of 5 numerators over the denominator “kilograms liveweight sold” (kg-LW) within indication for use at the lot and feedyard (yard) levels.

Indications for use are indicated to the right.

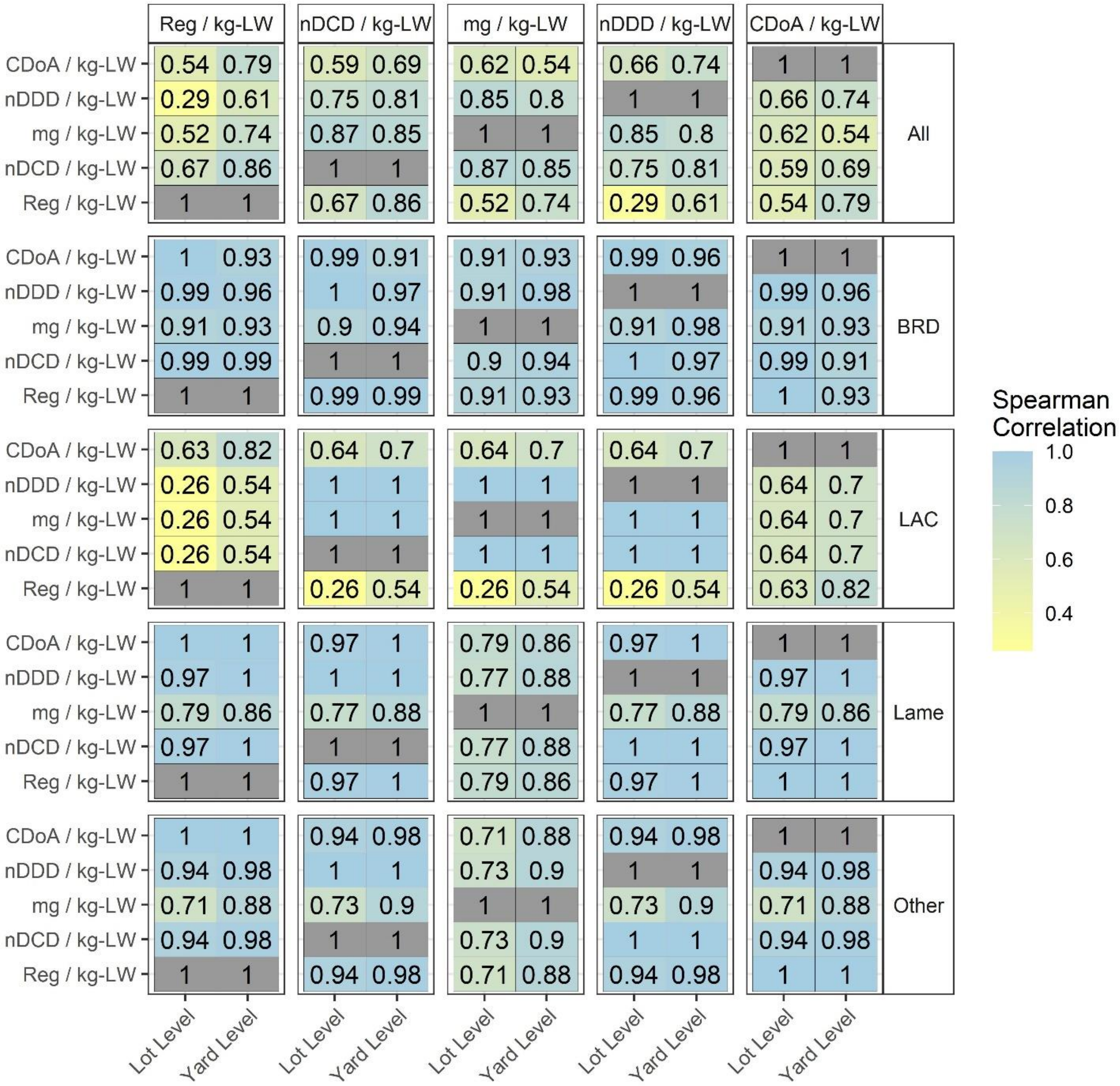

Supplement: Supplementary file 1 [file Data_Sheet_1.pdf]
